# Homozygous Familial Hypercholesterolemia: The Future Looks Brighter But Not for All∗

**DOI:** 10.1016/j.jacadv.2023.100324

**Published:** 2023-05-26

**Authors:** Frederick J. Raal

**Affiliations:** Faculty of Health Sciences, University of the Witwatersrand, Johannesburg, South Africa

**Keywords:** homozygous familial hypercholesterolemia, novel therapies, survival

**Figure:**
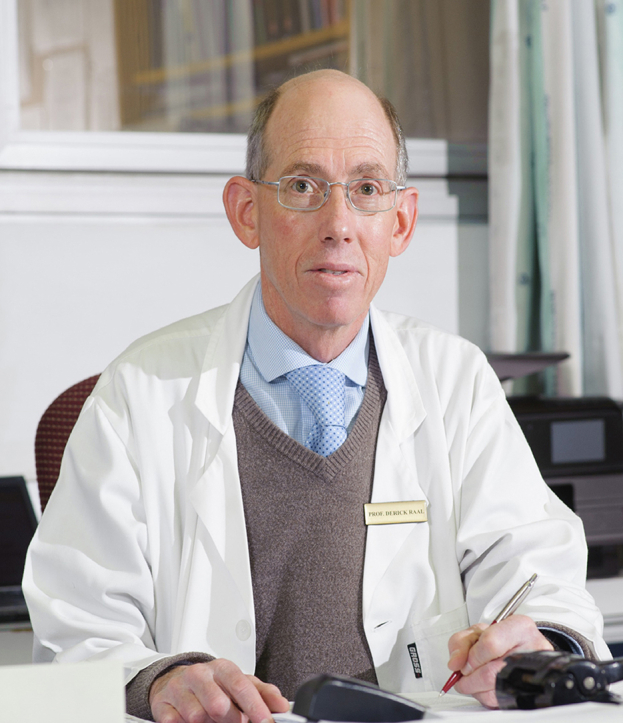

Homozygous familial hypercholesterolemia (HoFH) is a rare, but devastating, genetic disorder of lipid metabolism caused by mutations in both alleles of the low-density lipoprotein receptor (LDLR), or less frequently, apolipoprotein B, proprotein convertase subtilisin/kexin type 9, or the LDLR adapter protein.^1^^,^^2^ The condition affects approximately 1 in every 300,000 people worldwide. In some populations, such as the French Canadian, Afrikaners in South Africa, and Christian Lebanese, the prevalence of HoFH may be at least 10-fold higher due to founder effects.^1^ HoFH is characterized by extremely high plasma LDL-cholesterol (LDL-C) concentrations detectable at birth.^1^^,^^2^ These high plasma LDL-C levels lead to deposits of cholesterol in tendons, cutaneous tissues, and cardiovascular tissues, including the aortic root and valve. Severe and widespread atherosclerosis occurs in all major arterial beds and is often clinically evident at a young age.^1^^,^^2^ The severity of atherosclerosis tends to be proportional to the extent and duration of elevated LDL-C levels, calculated as the cholesterol-year score.^3^ Children as young as 4 years of age have suffered sudden death due to acute myocardial infarction, with nearly complete occlusion of the coronary artery or have required coronary artery bypass surgery before the age of 10 years.^4^ Although severe coronary atherosclerosis is the major cause of death, aortic stenosis has also been shown to be a key life-threatening complication in many individuals with HoFH and in young adults with HoFH, calcific aortic stenosis often requires aortic valve replacement.^5^ HoFH remains a difficult condition to treat but lipid-lowering therapy, with or without lipoprotein apheresis, should be instituted at diagnosis and as early as possible. Despite only reducing LDL-C modestly, statin therapy was shown to reduce cardiovascular and all-cause mortality in HoFH but LDL-C levels remained unacceptably high on statin therapy alone.^6^ Patients with HoFH therefore require multiple cholesterol-lowering drugs in addition to statins including ezetimibe, proprotein convertase subtilisin/kexin type 9-inhibitor therapy, lomitapide, and angiopoietin-like 3 directed therapies to lower LDL-C levels further.^7^, ^8^, ^9^ Lipoprotein apheresis may be used as an adjunct therapy for patients with HoFH who have an inadequate response to drug therapy alone but, even in combination with lipoprotein apheresis, treatments may be insufficient to lower LDL-C to recommended targets in these patients.

The paper by Brown et al^10^ in this issue of *JACC: Advances* reports on the diagnosis, management, and new treatment options of HoFH in Canada and highlights the improved survival in these high-risk patients. Although the study included only 48 subjects with HoFH, the long follow-up period of almost 30 years add credibility to their findings. The genetic testing done in the Canadian cohort shows that LDL-C levels in genetically confirmed HoFH subjects may be lower than expected and can overlap with LDL-C levels found in patients with heterozygous FH emphasizing that treatment needs to be aimed at the phenotype (LDL-C level) and not the genotype and that the diagnosis of HoFH needs to be considered in all patients with an untreated LDL-C of 400 mg/dL or greater.

A limitation of the study is that it is a single-country study and is not reflective of the current management of HoFH worldwide. Many HoFH patients remain undiagnosed and untreated or undertreated, particularly those living in low- or middle-income countries. In the HoFH International Clinical Collaborators registry, the event-free survival in HoFH was, on average, a decade shorter among HoFH patients managed in nonhigh-income countries.^5^

Of concern, not only in the Canadian cohort but worldwide, is that the diagnosis of HoFH is made too late as is the initiation of lipid-lowering therapy. As LDL-C levels of untreated HoFH subjects are elevated 4-fold, by the age of 10 years their arterial tree has been exposed to the same amount of LDL-C as a healthy 40-year-old, and by the age of 20 years to the same amount of LDL-C as a healthy octogenarian.^3^^,^^11^ As the mean age of diagnosis of HoFH in the Canadian cohort was 12 years, there was already a significant atherosclerotic burden in their HoFH cohort. Clearly, we need to diagnose and start treatment much earlier in order to delay the onset of atherosclerotic cardiovascular disease.

Overcoming the challenges of HoFH has been a long and difficult journey, but, as shown in the Canadian cohort, the treatment now available to us has converted HoFH from a lethal disorder in childhood to a more manageable dyslipidemia.

However, access to the new therapies, particularly novel therapies that work independently of LDLR function, such as lomitapide and the angiopoietin-like 3 inhibitor evinacumab, as well as to lipoprotein apheresis, is a major concern. We have a long way to go to ensure that all HoFH patients, particularly those living in less affluent countries, have access to these novel therapies that are highly effective in lowering LDL-C on top of statin therapy. This is essential and needs to be urgently addressed if we are to improve the quality of life of our HoFH patients in order to allow them to live healthier, longer lives.

## Funding support and author disclosures

Dr Raal has received advisory board fees and lecture fees from Amgen, Sanofi-Aventis, Regeneron Pharmaceuticals, Novartis, and LIB Therapeutics.
